# Successful Implementation of an Accelerated Recovery and Outpatient Total Joint Arthroplasty Program at a County Hospital

**DOI:** 10.5435/JAAOSGlobal-D-19-00110

**Published:** 2019-09-20

**Authors:** Blake J. Schultz, Nicole Segovia, Tiffany N. Castillo

**Affiliations:** From the Department of Orthopaedic Surgery, Stanford University Medical Center, Redwood City, CA (Dr. Schultz, Segovia, and Dr. Castillo); and the Department of Orthopaedics, Adult Total Joint Reconstruction, Santa Clara Valley Medical Center, San Jose, CA (Dr. Castillo).

## Abstract

Outpatient and accelerated recovery total joint arthroplasty (TJA) programs have become standard for private and academic practices. County hospitals traditionally serve patients with limited access to TJA and psychosocial factors which create challenges for accelerated recovery. The effectiveness of such programs at a county hospital has not been reported.

**Methods:**

In 2017, our county hospital implemented an accelerated recovery protocol for all TJA patients. This protocol consisted of standardized, preoperative medical and psychosocial optimization, perioperative spinal anesthesia, tranexamic acid and local infiltration analgesia use, postoperative emphasis on non-narcotic analgesia, and early mobilization. LOS, complications, disposition, and cost were compared between patients treated before and after protocol implementation.

**Results:**

In 15 months, 108 primary TJA patients were treated. Compared with the previous 108 TJA patients, LOS dropped from 3.4 to 1.6 days (*P* < 0.001), more patients discharged home (92% versus 72%, *P* < 0.001), average hospitalization and procedure-specific costs decreased 24.7% and 22.1%, respectively, and were significantly fewer complications (7% versus 21%, *P* = 0.007).

**Conclusions:**

Implementation of an accelerated recovery TJA program at a County Hospital is novel. This implementation requires careful patient selection and a coordinated multidisciplinary approach and is a safe and cost-effective method of delivering high-quality care to an underserved cohort.

Over the past several years, increasing adoption of accelerated recovery and outpatient total joint arthroplasty (TJA) programs was found. These programs have markedly reduced postoperative inpatient stays and cost in total hip arthroplasty (THA) and total knee arthroplasty (TKA), without increasing complication rates.^1,2,3,4,5^ However, critical to the success of these programs is that patients undergo careful risk stratification, medical optimization, and often resource-intensive preoperative and postoperative management.^6^ To date, limited literature investigating whether these protocols can be successfully implemented in more marginalized patient cohorts and resource-limited care settings is found.^7^ We sought to evaluate the outcomes of an accelerated recovery TJA program implemented at a California County Hospital.

In a County Hospital setting, the patient cohort often has a higher frequency of psychosocial factors affecting their health care access and outcomes.^8,9^ Our particular patient cohort often lacks access to routine health maintenance and medical optimization, has less reliable home support and transportation, higher substance abuse rates, and lower socioeconomic status (SES).

Although patients of lower SES have been shown to have a higher prevalence of hip and knee osteoarthritis,^8,10,11^ a disparity in rates of TJA among this cohort is found.^12,13^ When patients of lower SES are offered surgery, they often experience longer wait times to surgery, worse quality of life while waiting for surgery,^14^ worse functional outcomes, as well as decreased satisfaction and increased infection rates after TJA.^15,16,17,18,19,20^

Consequently, it is even more critical to adopt and implement standardized TJA care protocols for County Hospital patients, so that all medical and psychosocial risk factors are appropriately identified and addressed. Moreover, an ethical obligation not to deny the opportunity for outpatient and accelerated recovery protocols to these marginalized cohorts who already struggle with access to timely and high-quality care is found. Although all the aforementioned psychosocial factors can create additional challenges in implementing such programs, our hypothesis was that an accelerated recovery and outpatient TJA program could be safely and successfully implemented at our County Hospital.

## Methods

**Figure 1:**
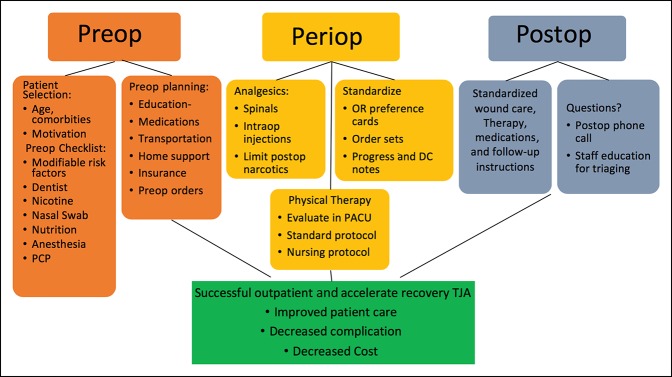
Flow chart illustrating different components of the protocol.

In August 2017, with the hire of an Adult Reconstruction Fellowship–trained orthopaedic surgeon, a new accelerated and outpatient TJA protocol was initiated at our County Hospital. The protocol was developed using the surgeon's experience and based on current literature and with the collaboration of a multidisciplinary team including nurses, operating room (OR) staff, physical therapists, case managers, social workers, pharmacists, information technologists, and hospital administrators. The protocol is similar to those that have been developed across the United States with a focus on preoperative medical optimization, patient education, early postoperative mobilization, standardized postoperative order sets, and early discharge planning^21,22^ (Figure 1).

A few unique characteristics pertaining to the patient cohort and hospital setting that our protocol had to take into account, including higher rates of non–English-speaking patients, housing insecurity, substance abuse, and infections, were found.^15,16,23^ We worked to educate referring providers about medical optimization through electronic outreach as well as a presentation at Medicine Grand Rounds and built our criteria into the electronic consult referral form including failure of Tylenol, NSAIDs, activity modification, and physical therapy to provide pain relief (Appendix 1, Supplemental Digital Content 1, http://links.lww.com/JG9/A56). Patients were required to be nonsmokers. Referrals to smoking cessation classes were placed as needed, and patients needed a negative nicotine test before surgery scheduling. There was no hard cutoff for body mass index (BMI) to have surgery was found, but patients were referred to nutrition counseling and bariatric surgeons as needed and had to make a true attempt at weight loss. Their BMI at every visit was recorded and given to the patient to help track. HgA1C was required to be <8.0 mg/dL and was preferred to be <7.5. Patient education and setting clear preoperative and postoperative expectations became standardized with handouts that were also translated into the three most common non-English languages in our clinic (ie, Spanish, Vietnamese, and Punjabi) (Appendix 1, Supplemental Digital Content 1, http://links.lww.com/JG9/A56). Clinic staff and providers were trained to employ the use of these handouts at all relevant clinic visits.^21^ Patients who had issues with housing, substance abuse, or other social issues were referred to a social worker to address their specific issues before scheduling surgery. Perioperatively, an emphasis on limiting narcotics by implementing the use of spinal anesthesia, intraoperative local infiltration analgesia, as well as a postoperative multimodal pain management protocol that is centered on the use of cryotherapy and standing non-narcotic analgesia with Tylenol and Toradol was found^24,25^ (Table 1). Peripheral nerve blocks were not routinely used. In addition, early postoperative mobilization with physical and occupational therapy on day of surgery, as well as social work and case management engagement, was instituted to facilitate safe and efficient discharge planning. Aspirin was the standard choice for deep vein thromboembolism (DVT) prophylaxis unless contraindicated or patients were on a different anticoagulant preoperatively.

**Table 1:**
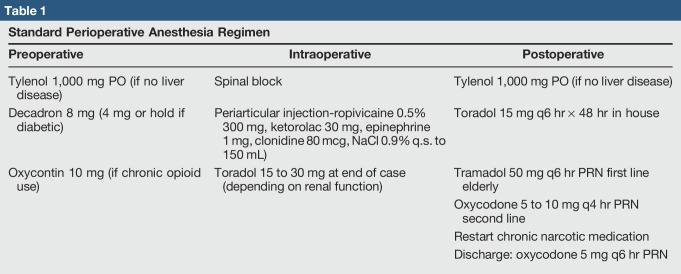
Standard Perioperative Anesthesia Regimen

Fifteen months after implementation of this new accelerated recovery TJA protocol, we received a waiver from institutional review board to conduct a quality improvement retrospective chart review of all the primary elective TKA and THA surgeries. Revision TJA and THA for femoral neck fractures were excluded due to the inability to implement the same preop and periop protocol in this cohort.^26^ The resulting 108 elective primary TJAs were compared with the 108 elective primary TJAs done over the 20 months just before our new TJA protocol implementation. In cases where a patient had more than one primary TJA done, they were treated as separate cases.

Previously, TJA was done by five board-certified orthopaedic surgeons, one of whom had a combined Trauma and Adult Reconstruction Fellowship training. After protocol implementation, all elective TJA was done by a single Adult Reconstruction Fellowship–trained orthopaedic surgeon. For THA before and after protocol, all surgeons used a posterior approach and press-fit femoral and acetabular components. For TKA, all surgeons used a standard medial parapatellar approach, and the surgeons before the new protocol used posterior stabilized components while the arthroplasty fellowship–trained surgeon used a cruciate retaining femoral implant and ultracongruent or medial congruent polyethylene. Before the new TJA protocol, patients had general anesthesia, peripheral nerve blocks, foley placement, did not have local infiltration analgesia, variable use of tranexamic acid, staple skin closure, physical therapy on POD 1, and lovenox for DVT prophylaxis.

Primary outcome measures included acute medical and surgical complications, LOS, discharge destination (ie, skilled nursing facility [SNF], home, and acute rehab), unplanned readmission and return to the OR within 90 days, and cost of both the procedure and total hospital stay. Patient demographic information including age, sex, American Society of Anesthesiology (ASA) classification, and insurance type was also collected. Complications were tracked and categorized by reviewing surgical notes of the operating surgeon and through chart review by an orthopaedic surgery resident of all operative, progress, emergency department and clinic notes, and laboratory test results. All patients had at least 90 days of follow-up. Cost data were acquired from the hospital billing department based on CPT codes for primary TKA and THA. The cost of the procedure and the total hospital stay were detailed and adjusted for inflation to represent $2018.

Chi-square tests and the Fisher exact test were used for categorical variables and Student *t*-tests for continuous variables. All statistical analyses were completed in RStudio version 1.1.456 using a two-sided level of significance of 0.05.

## Results

### Patient Demographics

In total, 216 elective primary TJAs over a 35-month period were reviewed. The 108 TJAs in the preprotocol group consisted of 29 THAs and 79 TKAs. The 108 TJA protocol patients consisted of 44 THAs and 64 TKAs (Table 2). Markedly more THAs done in the protocol group (*P* = 0.044) were found. Eight patients who had a TJA both before and after protocol were found. Patient age, BMI, and insurance type (ie, Medicare, county medical, and other) were not markedly different between groups (Tables 2 and 3). Markedly more patients in the preprotocol group had an ASA class of three compared with patients in the protocol group (38% versus 3%, *P* < 0.001).

**Table 2:**
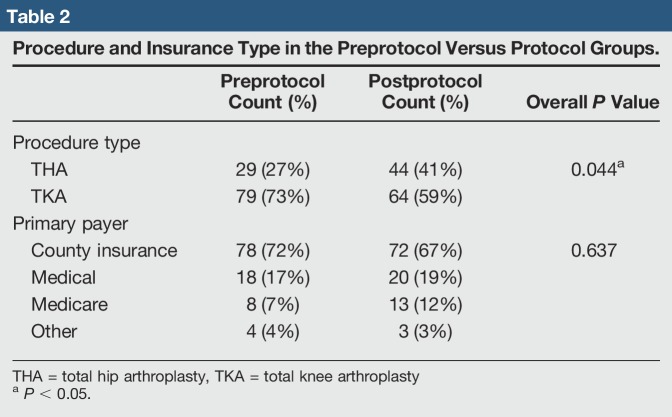
Procedure and Insurance Type in the Preprotocol Versus Protocol Groups.

**Table 3:**
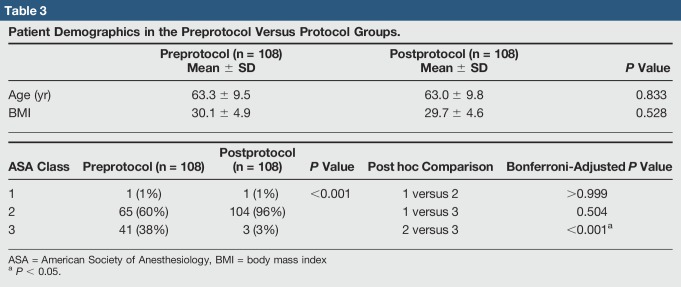
Patient Demographics in the Preprotocol Versus Protocol Groups.

### Length of Stay and Disposition

Length of stay in the protocol group dropped from 3.4 (SD = 1.6) to 1.6 (SD = 1.1) days for all TJA (*P* < 0.001) (Table 4). For THA specifically, LOS dropped from 3.4 to 1.4 days and for TKA from 3.4 to 1.7 days (both *P* < 0.001). Seven outpatient (same day) TJA (five THA and two TKA) home discharges, all in the protocol group, were found. Significantly more patients in the protocol group were discharged home (versus SNF or rehab) than patients in the preprotocol group (92% versus 72%, *P* < 0.001).

**Table 4:**
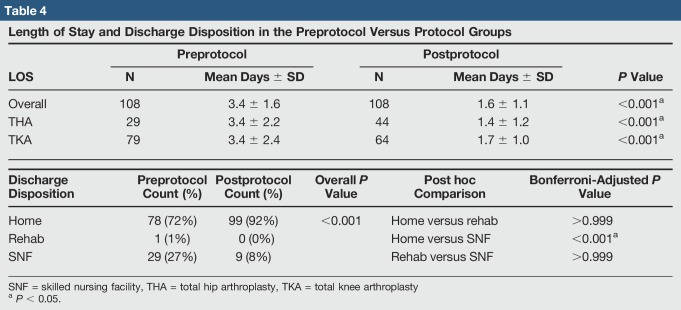
Length of Stay and Discharge Disposition in the Preprotocol Versus Protocol Groups

### 90-Day Readmissions and Complications

Protocol patients experienced significantly fewer complications overall compared with preprotocol patients (7% versus 21%, *P* = 0.007), specifically with fewer acute medical complications (4% versus 12%, *P* = 0.040) (Table 5). Superficial wound complications, deep wound complications, acute surgical complications, unplanned 90-day readmission, and return to OR within 90 days did not differ between groups.

**Table 5:**
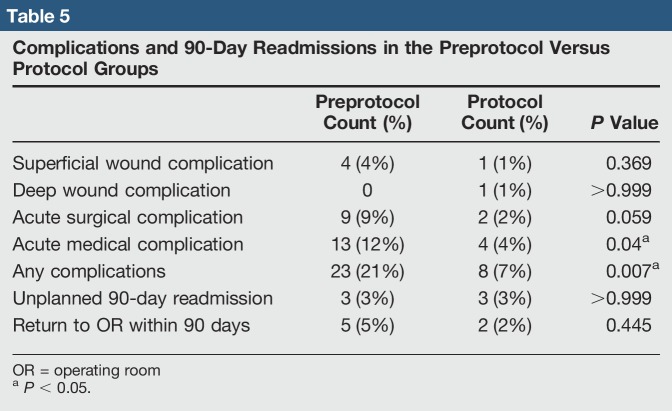
Complications and 90-Day Readmissions in the Preprotocol Versus Protocol Groups

Twenty-six total complications in the preprotocol group, with three unplanned readmissions (one medical and two surgical) and five returns to the OR within 90 days, were found (Table 4). Seven of the complications were acute surgical complications including intraoperative acetabular fracture, intraoperative patella fracture (medial facet fracture during patella preparation that did not involve extensor mechanism, the fragment was excised, the patella was not resurfaced, and ROM was restricted to <45° of flexion for 1 month), two knee arthrofibrosis requiring manipulation under anesthesia (POD 35 and POD 60), intraoperative MCL injury requiring acute repair, postoperative TKA hemarthrosis that was aspirated on the floor POD 1 and resolved without additional intervention, and one postoperative transient quadriceps paresis believed to be related to the nerve block or tourniquet that required transfer to PMR service before discharge, with eventual resolution. Six superficial wound complications were found; three were treated with wound care and close clinic follow-up (including one with an incisional wound vac), and three returned to the OR for superficial irrigation and débridement (two on POD 7 and one on POD 56). No reported deep infections were found. Thirteen acute medical complications including five patients with acute postoperative anemia requiring blood transfusion, three with DVT picked up on ultrasounds ordered for symptomatic examination, one with pulmonary embolism, one urinary retention requiring foley catheter reinsertion, one with postoperative hyponatremia requiring an additional day in the hospital, one with syncope POD 1 requiring transfer to the medical intensive care unit, and one with new dysphagia POD 5 requiring readmission to the medicine service were found.

In the protocol group, seven total complications, with three unplanned readmissions (one surgical and two medical) and two returns to the OR, were found. Two surgical complications, including one intraoperative patella fracture (small superior pole fragment that was excised without change in postop care or recovery) and one knee arthrofibrosis requiring manipulation under anesthesia (POD 89), were found. One superficial wound complication that was a suture reaction requiring no intervention was found. One deep infection in a THA patient who contracted an acute periprosthetic joint infection after electing to serve jail time 3 weeks postop and being roomed with an inmate with an active staph hand infection was found. Four medical complications, including one pulmonary embolism, one acute postoperative anemia requiring blood transfusion, one hyponatremia requiring extended hospital stay, and one patient with syncope on POD 40 requiring readmission to the medicine service and workup attributed to patient's known pre-existing vertebrobasilar insufficiency, were found.

### Costs

The average total hospitalization cost for TJA (adjusted to $2018) in the protocol group dropped 24.7% from $100,749 (SD = 19,211) to $75,911 (SD = 15,334) (*P* < 0.001) (Table 6). For THA specifically, total hospitalization cost dropped 26.1% from $101,057 to $74,680 and for TKA dropped 23.9% from $100,685 to $76,758 (both *P* < 0.001). The average procedure cost for TJA dropped 22.1% from $10,206 (SD = 2,675) to $7,950.23 (SD = 140.61) (*P* < 0.001). For THA specifically, average procedure cost dropped 19.1% from $9,675 to $7,943 and for TKA dropped 23.6% from $10,400 to $7,955 (both *P* < 0.001). In addition to the overall decrease in mean cost, a notable decrease in the SD of procedure costs across TJA was found.

**Table 6:**
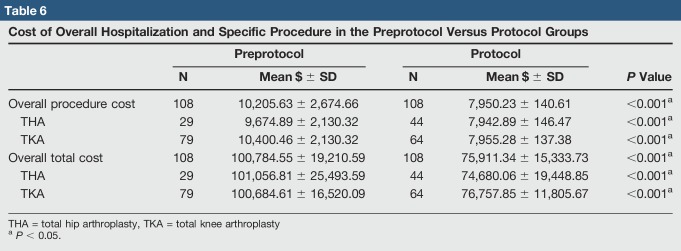
Cost of Overall Hospitalization and Specific Procedure in the Preprotocol Versus Protocol Groups

For the eight patients who had a TJA in both preprotocol and protocol groups, the results were similar to the overall group data. The average LOS dropped from 3.8 to 1.0 day (*P* = 0.004) (Table 7). In the preprotocol group, six patients discharged home and two to SNF, and two complications were found, both acute medical complications (Table 8). In the protocol group, all patients discharged home, and no complications were found. Average procedure and overall hospitalization cost were also less, but only the difference in overall cost was statistically significant (*P* = 0.134 and *P* = 0.010, respectively) (Table 9).

**Table 7:**
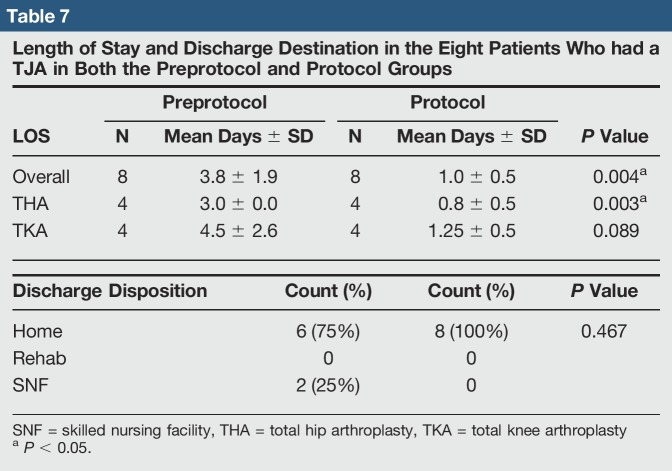
Length of Stay and Discharge Destination in the Eight Patients Who had a TJA in Both the Preprotocol and Protocol Groups

**Table 8:**
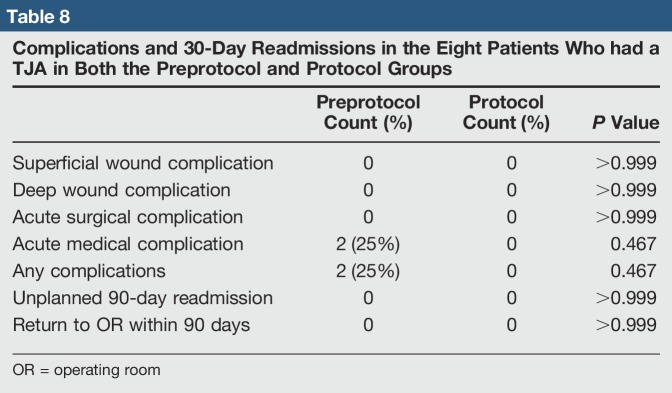
Complications and 30-Day Readmissions in the Eight Patients Who had a TJA in Both the Preprotocol and Protocol Groups

**Table 9:**
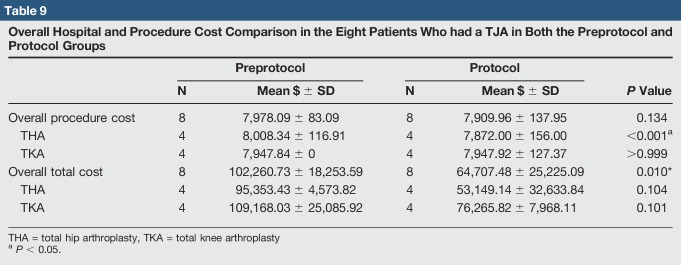
Overall Hospital and Procedure Cost Comparison in the Eight Patients Who had a TJA in Both the Preprotocol and Protocol Groups

## Discussion

Numerous studies have shown the safety, efficiency, and cost-saving potential of accelerated and outpatient TJA in select cohorts.^1,2,3,4,5^ Although a County Hospital patient cohort tends to have a higher rate of psychosocial and medical risk factors than traditionally studied TJA cohorts, our results suggest that an accelerated recovery and outpatient TJA protocol can be implemented safely and with notable cost-saving potential.

The demographics of the patients between groups were mostly similar except for the lower rate of ASA 3 classification in the protocol group. Although ASA is not a perfect surrogate for health status, it does give a general sense of a patient's overall health. The difference in ASA between the protocol and preprotocol group could be seen as a limitation of the study or, alternatively, could be a reflection of the use of more standardized patient selection protocol and close work with primary care providers on preoperative medical optimization.^22^ The same county hospital population was treated in both groups, so there is no reason to believe there is a difference in the overall health status of patients presenting to the orthopedic clinic between groups would be present. In addition, although ASA been shown to be an accurate predictor of postoperative discharge location and readmission rate,^27,28^ the literature is not clear on the association between ASA and LOS,^28,29^ making our results relevant regardless of the difference in ASA status. With regard to the increased proportion of THA done in the protocol group, this is likely a reflection of the increased comfort of the arthroplasty fellowship-trained surgeon in doing THA compared with nonarthroplasty-trained surgeons. A national trend for primary THA to be done by fellowship-trained arthroplasty surgeons, which is reflected in this trend at our institution, is found.^30^ A multivariable linear regression analysis was run, concluding that the increase in the percentage of THA from the pr-protocol to the protocol group did not markedly affect any of the length of stay or cost outcomes (all *P* < 0.001) (Appendix 2, Supplemental Digital Content 2, http://links.lww.com/JG9/A57).

The decrease in average LOS from 3.4 to 1.6 days in the protocol group brought our hospital below the national average of 3 days^30,31,32,33^ and is similar improvement compared with other accelerated recovery TJA programs.^7,21,22,34^ The following implies that an accelerated recovery program can be as effective at County Hospital as they are in other high-volume joint centers. The seven same-day TJA cases (6.48%) represents an area of growing improvement in the new TJA protocol, and this rate continued to increase after the formal study period. Successful outpatient TJA relies on a strong coordinated social support system,^35^ which remains an ongoing challenge in our County Hospital cohort.

The markedly lower overall complication and acute medical complication rates demonstrate a notable improvement in the quality of TJA care delivered. The etiology of these results is likely multifactorial, but is undoubtedly related to the focus on extensive preoperative medical and psychosocial optimization, coordination of care, patient education, and the standardization of perioperative and postoperative care protocols. The fact that no difference was found in the surgical or wound complications, 90-day readmissions, or return to OR again demonstrates the safety of our accelerated recovery TJA protocol.

Decreasing complications and LOS represent a tremendous cost-saving opportunity for our County Hospital,^34,36-38^ which was shown in the 24.7% change in average hospital cost per patient. The average cost per procedure also decreased 22.1%, with a very small SD, which is likely the result of standardization of surgical room equipment, implants, and protocols. Our study likely underestimates the total cost savings because we did not include readmission costs, which can be extremely expensive, especially if they require subsequent surgery.^39^ In addition, we did not include any potential savings incurred from the notable decrease in discharge SNFs. Postacute care has been shown to account for 36% to 55% of total costs associated with an episode of TJA care.^40,41^ Keeping in mind that Medicare patients, which make up a large percentage of our County Hospital cohort, have markedly longer stays in SNFs after TJA than patients covered by Health Maintenance Organizations or Preferred Provider Organizations,^42^ increasing discharges to home instead of SNFs represents another potential area of notable cost savings that was not accounted for in our data.

The strengths of our study include that it is as large, comprehensive review of a homogenous, consecutive patient cohort was found. As with all retrospective studies that are dependent on the accuracy of chart review, potential for missing postoperative complications is found. However, our chart review, specifically in the protocol group, was checked against the operating surgeon's personal notes to ensure accuracy. Readmissions to other hospitals in the preprotocol group could have been missed since we only had access to the records at our County Hospital. However, since a large percentage of our patients rely on care at our institution secondary to insurance limitations, they are more likely to represent to our hospital by default if any complications are found.

A potential limitation of our study is that the new TJA protocol was implemented by a single surgeon, and although this ensured the standardization of intraop techniques and periop care, the following may not have the same generalizability if implemented by nonarthroplasty-trained surgeons. Higher surgeon volume is known to be correlated with decreased infection, readmission rates, and LOS and increased likelihood of being discharged home.^43^ Before the protocol, TJA was done by five different low-volume TJA surgeons, compared with after the protocol where all TJA were done by a single high-volume TJA surgeon. Using high-volume surgeons to help standardize the procedure is a common component of implementing accelerated recovery TJA programs; so, although readers should be aware of this difference, it does not invalidate the results. Because the protocol implements many perioperative changes (eg, preoperative optimization protocols, pain management, and early postoperative therapy.), it is difficult to identify confounding variables. As the program continues to evolve, a future area of study will be found. Another limitation is our relatively short-term follow-up. To include as many patients as possible in this newly implemented protocol, complication data were only reviewed up to 90 days postoperatively for the latest patients in the protocol group. Longer follow-up will be necessary to ensure that no increased mid-term or long-term complications rates among this cohort are found. Finally, we did not have complete data to compare any patient-reported or functional outcomes between groups, which will be an important area of focus for future research in our patient cohort.

## Conclusion

Many successful outpatient and accelerated TJA programs have been implemented across the country, however implementation in a County Hospital system is novel. As with any successful TJA program, the standardized implementation of contemporary evidence-based TJA care is critical.^21,22^ Having the coordination and buy-in of a multidisciplinary team, meticulous tracking of results, and support from the administration and a dedicated program champion is important.^21,22^ It is also essential that the refinement of the program be an iterative process with frequent input from all stakeholders. Although a County Hospital setting and patient cohort offer a unique set of challenges, our data suggest that they can be adequately addressed to deliver safe and cost-effective TJA care to traditionally underserved patients (Appendix 1, Supplemental Digital Content 1, http://links.lww.com/JG9/A56, and Appendix 2, Supplemental Digital Content 2, http://links.lww.com/JG9/A57).
